# Chronic Intermittent Fasting Improves Cognitive Functions and Brain Structures in Mice

**DOI:** 10.1371/journal.pone.0066069

**Published:** 2013-06-03

**Authors:** Liaoliao Li, Zhi Wang, Zhiyi Zuo

**Affiliations:** 1 Department of Anesthesiology, University of Virginia, Charlottesville, Virginia, United States of America; 2 Department of Anesthesiology, Sun Yat-Sen Memory Hospital, Sun Yat-Sen University, Guangzhou, Guangdong, China; Massachusetts General Hospital, United States of America

## Abstract

Obesity is a major health issue. Obesity started from teenagers has become a major health concern in recent years. Intermittent fasting increases the life span. However, it is not known whether obesity and intermittent fasting affect brain functions and structures before brain aging. Here, we subjected 7-week old CD-1 wild type male mice to intermittent (alternate-day) fasting or high fat diet (45% caloric supplied by fat) for 11 months. Mice on intermittent fasting had better learning and memory assessed by the Barnes maze and fear conditioning, thicker CA1 pyramidal cell layer, higher expression of drebrin, a dendritic protein, and lower oxidative stress than mice that had free access to regular diet (control mice). Mice fed with high fat diet was obese and with hyperlipidemia. They also had poorer exercise tolerance. However, these obese mice did not present significant learning and memory impairment or changes in brain structures or oxidative stress compared with control mice. These results suggest that intermittent fasting improves brain functions and structures and that high fat diet feeding started early in life does not cause significant changes in brain functions and structures in obese middle-aged animals.

## Introduction

Obesity prevalence has been increased over the years. About one third of American adults and 20% teenagers now are obese [1], [2]. High fat diet has been considered as a significant contributing factor for the pandemic of obesity in the USA [3]. It is known that obesity and its associated metabolic disturbance including hyperlipidemia have been identified as risk factors for cardiovascular diseases, diabetes and many other diseases [4]. However, very little is known about the effects of obesity on brain functions and structures.

Dietary restriction increases average and maximum life span [5], [6]. It also decreases aging-related learning and memory impairments in animals and human [7], [8]. A recent study showed that caloric restriction attenuates aging-related brain atrophy in monkeys [6]. In addition, dietary restriction can stabilize the expression of synaptic protein expression to avoid aging-related changes [9]. These results suggest that dietary restriction attenuates the brain aging process. There are two methods of dietary restriction. One method is to provide a food allotment that is about 60 – 70% of that consumed by control animals with ad libitum food. This method is called caloric restriction and will usually result in significant decrease of body weight [10]. The second method involves subjecting animals to intermittent (alternate-day) fasting. Intermittent fasting is known to decrease food intake and body weight over time [11]. Interestingly, intermittent fasting but not caloric restriction for 20 weeks increases hippocampal neuron tolerance to excitotoxic stress in mice, suggesting neuroprotective effects of intermittent fasting [10].

Despite of the apparent significance, the effects of early onset and long-term obesity and intermittent fasting on learning and memory and brain structures have not been examined in the middle aged animals or humans (before obvious aging-related processes have started). To study these effects, we fed young mice with high fat-diet or subjected them to intermittent fasting for 11 months. Their leaning, memory, brain biochemical and structural changes were determined. We focused on measuring brain oxidative stress indices because oxidative stress is known to induce cell injury and impairment of cognitive functions [12], [13]. These studies simulate human teenager obesity that continues to the adulthood and also examine the protective effects of intermittent fasting on the brain under this clinically relevant condition.

## Results

Total 15, 19 and 15 mice were assigned to the control, intermittent fasting and high fat-diet feeding groups. Each cage hosted 3 to 4 mice during the 11-month feeding period. Fifteen mice from each group survived till the end of the feeding period. There was no significant difference in the mortality rates among the three groups. The 4 mice that died in the intermittent fasting group were the lightest among their cage-mates from the beginning of the feeding protocol till their death.

**Figure 1:**
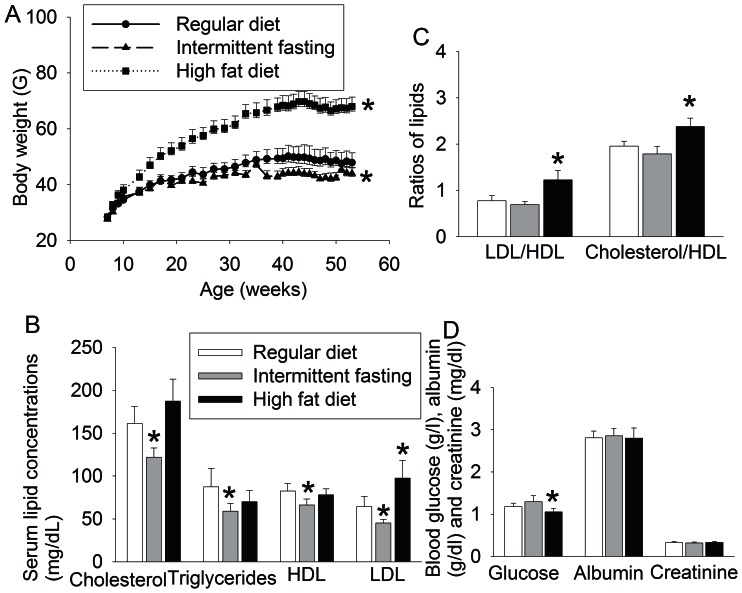
The effects of various feeding protocols on body weights and blood chemicals. Seven-week old male mice had free access to regular chow or high fat diet or were allowed to have free access to regular chow every other day (intermittent fasting) for 11 months. Their growth curves are presented in panel A. Their blood was drawn at the end of the 11-month feeding to measure lipid profiles (panels B and C), glucose, albumin and creatinine (panel D). Results are means ± S.E.M (n = 12 – 15). * P<0.05 compared with mice on regular chow ad libitum. HDL: high density lipoprotein; LDL: low density lipoprotein.

Mice in all three groups increased their weights over time. Intermittent fasting had a significant effect on body weights [F(1, 25) = 4.644, P = 0.041]. Mice on intermittent fasting were lighter than control mice since they had been on corresponding feeding protocols for 37 weeks. Mice on high fat diet were heavier than control mice since they were on corresponding feeding protocol for 13 weeks. At the end of 11-month feeding protocol, mice on high fat diet were ∼45% heavier than the control mice. High fat diet had a very significant effect on the body weights [F(1, 25)  = 111.119, P<0.001] (Fig. 1). Intermittent fasting significantly decreased blood cholesterol, triglycerides, high density lipoproteins (HDL) and low density lipoproteins (LDL) but did not affect the ratio of LDL/HDL and cholesterol/HDL in the blood. Intermittent fasting also did not affect the fasting blood levels of albumin, creatinine and glucose. High fat diet feeding significantly increased LDL and the ratio of LDL/HDL and cholesterol/HDL. Mice on high fat diet also had decreased fasting blood glucose levels. Blood cholesterol in mice fed with high fat diet trended to be higher than that in the control mice (P = 0.124) (Fig. 1).

**Figure 2:**
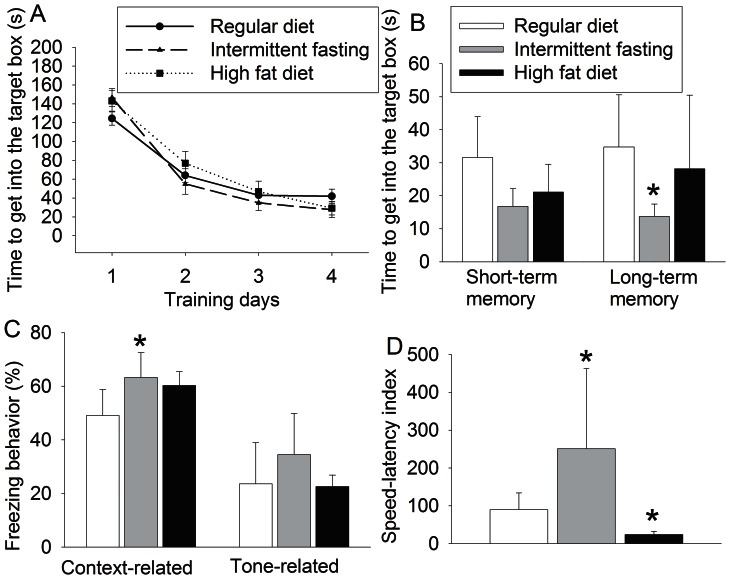
The effects of various feeding protocols on leaning and memory. Seven-week old male mice had free access to regular chow or high fat diet or were allowed to have free access to regular chow every other day (intermittent fasting) for 11 months. They were then subjected to Barnes maze, fear conditioning and rotarod tests. The results in the training sessions and memory phase of the Barnes maze are presented in panels A and B. The fear conditioning and rotarod results are shown in panels C and D, respectively. Results are means ± S.E.M (n = 15 – 35). * P<0.05 compared with mice on regular chow *ad libitum*.

The time for mice in all three groups to get into the target box in the Barnes maze test became shorter with increased training sessions. Neither intermittent fasting [F(1, 48) = 0.0555, P = 0.815] nor high fat diet [F(1, 48) = 0.313, P = 0.579] had a significant effect on the time for the mice to get into the target box during the training sessions. The time for the mice in the intermittent fasting group to get into the target box at 1 day after the training sessions trended to be shorter than that of control mice (P = 0.132). The time difference was significant when the test was done at 7 days after the training sessions. The mice in the intermittent fasting group also had increased context-related freezing behavior (Fig. 2). These results suggest that mice in the intermittent fasting group have better learning and memory than control mice. High fat feeding did not affect the performance of mice in the Barnes maze and fear conditioning compared with control mice (Fig. 2). Intermittent fasting increased the exercise tolerance tested on rotarod but high fat diet significantly worsened the exercise tolerance of the mice (Fig. 2).

**Figure 3:**
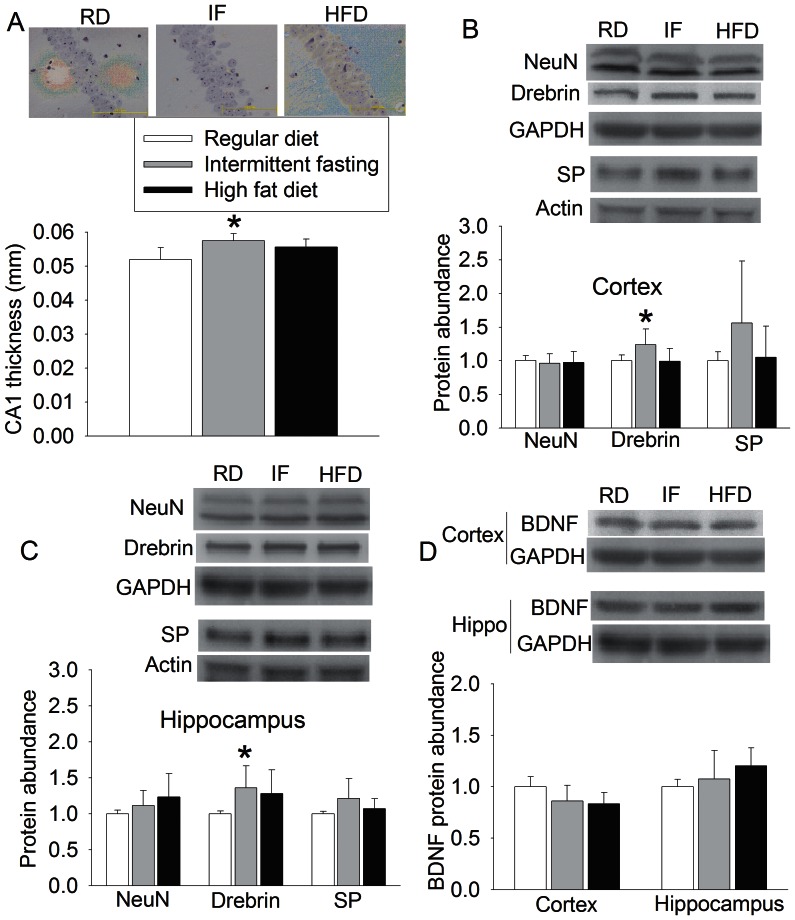
The effects of various feeding protocols on CA1 pyramidal cell layer thickness, neuron-specific proteins and brain derived neurotrophic factor (BDNF) in the brain. Seven-week old male mice had free access to regular chow or high fat diet or were allowed to have free access to regular chow every other day (intermittent fasting) for 11 months. Their brains were then harvested to measure CA1 pyramidal cell layer thickness (panel A) and expression of NeuN, drebrin and synaptophysin in the cerebral cortex (panel B) and hippocampus (panel C) as well as the BDNF levels in the cerebral cortex and hippocampus (panel D). The protein abundance results in each mouse were normalized by the mean value of the corresponding protein in the regular diet-fed mice. Results are means ± S.E.M (n = 7 – 12). * P<0.05 compared with mice on regular diet ad libitum. GAPDH: glyceraldehydes 3-phosphate dehydrogenase; Hippo: hippocampus; SP: synaptophysin; RD: regular diet; IF: intermittent fasting; HFD: high fat diet.

Thickness of pyramidal cell layer of CA1 region was used as an indicator to measure the changes of gross brain structures caused by intermittent fasting and high fat diet. Mice in intermittent fasting group had thicker CA1 pyramidal cell layer than control mice. They also had increased drebrin, a dendritic protein, in the cerebral cortex and hippocampus. The expression of synaptophysin, a synaptic protein, trended to be increased in the cerebral cortex (P = 0.157) and hippocampus (P = 0.082). High fat diet feeding did not affect the thickness of CA1 pyramidal cell layer and the expression of drebrin and synaptophysin. The anti-brain derived neurotrophic factor (BDNF) antibody detected a protein band at ∼15 kDa, which corresponding to the mature/active BDNF molecular weight. Neither intermittent fasting nor high fat diet affected the levels of this mature BDNF in the cerebral cortex or hippocampus (Fig. 3).

**Figure 4:**
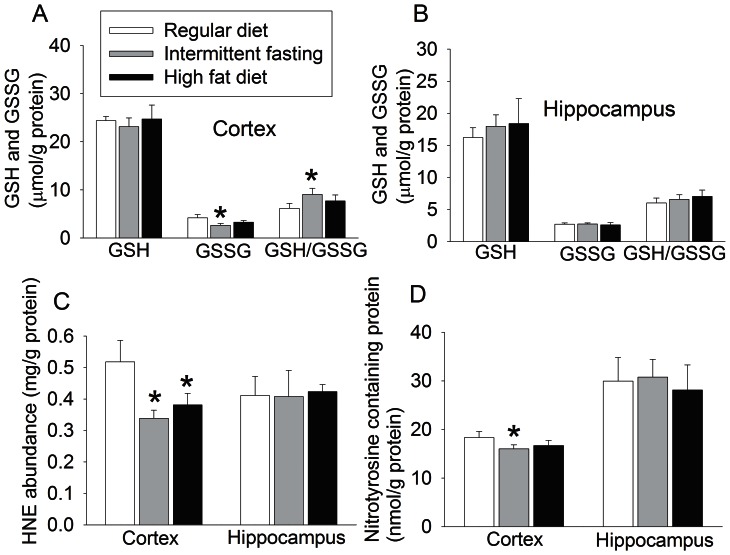
The effects of various feeding protocols on oxidative stress status in the brain. Seven-week old male mice had free access to regular chow or high fat diet or were allowed to have free access to regular chow every other day (intermittent fasting) for 11 months. Their brains were then harvested to measure glutathione (GSH), glutathione disulfide (GSSG), 4-hydroxy-2-nonenal (HNE) and nitrotyrosine containing proteins. The levels of GSH and GSSG and ratio of GSH/GSSG in the cerebral cortex and hippocampus are presented in panels A and B, respectively. The levels of HNE and nitrotyrosine containing proteins in the cerebral cortex and hippocampus are shown in the panels C and D. Results are means ± S.E.M (n = 8). * P<0.05 compared with mice on regular chow *ad libitum*.

Intermittent fasting did not affect the glutathione levels in the cerebral cortex and hippocampus. The glutathione disulfide (GSSG) level in the cerebral cortex was significantly reduced by intermittent fasting. This effect resulted in a significant increase in the ratio of glutathione/GSSG, suggesting a relatively higher reduction status in this brain region of mice in the intermittent fasting group. Consistent with this finding, intermittent fasting decreased the level of 4-hydroxy-2-nonenal (HNE) and nitrotyrosine containing proteins, two indicators for oxidative stress, in the cerebral cortex. Intermittent fasting and high fat diet did not affect the glutathione, GSSG, HNE and nitrotyrosine containing proteins in the hippocampus. High fat diet feeding appeared to significantly reduced HNE levels in the cerebral cortex but did not affect the levels of glutathione, GSSG and nitrotyrosine containing proteins in the cerebral cortex (Fig. 4).

## Discussion

Our results showed that intermittent fasting improved learning and memory as measured by Barnes maze and fear conditioning. Intermittent fasting also increased the thickness of CA1 pyramidal cell layer and drebrin expression in the hippocampus. These results suggest that intermittent fasting improves brain functions and structures. To determine possible mechanisms for this protection, we measured BDNF that was found to be increased in the brain of Huntingtin mutant mice after caloric restriction [14]. We did not observed any differences in mature BDNF expression in the intermittent fasting mice compared with control mice. However, intermittent fasting increased the ratio of glutathione/GSSG and reduced HNE and nitrotyrosine containing proteins, two oxidative stress indices, in the cerebral cortex. These results suggest that intermittent fasting reduces oxidative stress in the brain. Since oxidative stress is a known factor contributing to brain aging [15] and can induce cell injury and impairment of learning and memory [12], [13], our results indicate that reduced oxidative stress may be a mechanism for the improved brain functions and structures in the intermittent fasting mice.

A surprising result is that mice fed with high fat diet did not have impaired learning and memory and changed CA1 pyramidal cell layer thickness and expression of drebrin and synaptophysin when compared to control mice. Associated with this finding is no change in the expression of oxidative stress indices. However, mice fed with high fat diet were obese as reflected by their body weights that were about 45% higher than those of control mice. High fat diet feeding also resulted in hyperlipidemia. Associated with these findings was worse exercise tolerance as reflected by poorer performance on the rotarod. Together, these results suggest that high fat diet feeding induced mouse obesity that simulates human obesity very well and these obese middle-aged mice did not have impaired learning and memory. Their brain structures may also not be affected yet. We used multifold paradigms and parameters to measure learning, memory and brain structural changes. It is still possible that minor brain structural and functional alternations in these obese mice may not be revealed by these methods.

We fed 7-week old mice with high fat diet for 11 months. They were about 13 – 14 months when they were subjected to learning and memory tests. We chose this protocol to study the effects of long-term obesity started in teenagers on the learning, memory and brain structures of middle aged animals. There are no data on this issue in the literature. However, this issue has become increasingly important as teenager obesity is increasing rapidly [2]. The negative findings in our study suggest that significantly harmful effects on the brain have not occurred yet in the obese middle aged mice. However, the effects of long-term obesity on aging-related brain changes are not clear yet. Future studies are needed to address this issue by using obese elderly animals.

We observed that hippocampus-related learning and memory were improved in the mice with intermittent fasting because their performance in the context-related learning and memory in the fear conditioning test was better than control mice. Mice with intermittent fasting also had thicker CA1 pyramidal cell layer and higher expression of drebrin in the hippocampus. These structural improvements in hippocampus may have contributed to the better performance of these mice in the hippocampus-dependent learning and memory. However, the oxidative stress as measured by HNE and nitrotyrosine containing proteins was not decreased by intermittent fasting. This finding is different from that in the cerebral cortex where our results indicate functional and structural improvement and reduced oxidative stress. These findings suggest that mechanisms other than reduction of oxidative stress are involved in the improvement of hippocampal functions and structures or that our measurement of oxidative stress does not really reflect its status in the hippocampus. We measured HNE and nitrotyrosine containing proteins in the samples prepared from the whole hippocampus. Region-specific oxidative stress status will not be identified by this method. In addition to oxidative stress, various other insults/factors, such as immune and inflammatory responses, can harm brain functions and structures [16], [17].

Our results showed that intermittent fasting reduced oxidative stress in the brain. We have not investigated how intermittent fasting achieves this effect. However, it is known that mitochondria are a major organelle to produce oxidants and that intermittent fasting can induce metabolic reprogramming [14], [18]. Nevertheless, the possibility that intermittent fasting can reduce the production of oxidants via metabolic reprogramming in the mitochondria will need to be determined in future studies.

In summary, we have shown that intermittent fasting reduced oxidative stress in the brain and improved brain function and structures in mice. High fat diet feeding for 11 months started at the age of 7-weeks induced hyperlipidemia but did not affect the brain function and structures in mice.

## Materials and Methods

### Animal groups

The animal protocol was approved by the Institutional Animal Care and Use Committee of the University of Virginia (Charlottesville, VA, USA). All animal experiments were carried out in accordance with the National Institutes of Health Guide for the Care and Use of Laboratory Animals (NIH publications number 80–23) revised in 1996.

Seven week-old male CD-1 wild type mice were randomly divided into control, intermittent fasting and high fat diet group. Mice in control group were allowed to access regular chow (rodent diet #5010, Ralston-Purina Co., St. Louis, MO) freely. Mice in the intermittent fasting group were allowed free access to regular chow every other day and no food on the alternate day. Mice in high-fat diet had free access to high-fat food (45% caloric supplied by fat) (D12451, Research Diets, Inc., New Brunswick, NJ). Mice in the three groups were on the corresponding feeding protocols for 11 months. All mice had free access to water all the time. The regular chow has the following energy composition: 29% from proteins, 58% from carbohydrate and 13% from fat. The energy composition for the high fat diet is: 20% from proteins, 35% from carbohydrate and 45% from fat. Of note, the protein content of the regular chow and the high fat diet is 24.6% and 24%, respectively, by weight. Thus, these two diets have similar protein content. We choose to use 45% fat diet because this composition represents a typical Western diet [19].

### Barnes maze

After being on the corresponding feeding protocols for 11 months, mice were then subjected to Barnes maze as we described before [20]. Barnes maze is designed to test spatial learning and memory. Mice were placed in the middle of a circular platform with 20 equally spaced holes (SD Instruments, San Diego, CA). One of the holes was connected to a larger chamber that was called target box. Mice were encouraged to get into this box by aversive noise (85 dB) and bright light (200 W) shed on the platform. Mice went through a spatial acquisition phase that took 4 days with 4 trials per day and 15 min between each trial. Mice then went through the reference memory phase to test the short-term retention on day 5 and long-term retention on day 12. One test on each of these two days was performed. No trial was performed during the period from day 5 to day 12. The latency to get into the target box during each trial was recorded.

### Fear conditioning

One week after the long-term memory test of Barnes maze, mice were subjected to fear conditioning test. Mice were placed in a test chamber wiped with 70% alcohol and subjected to 3 tone-foot shock pairings (tone: 2000 Hz, 90 db, 30 s; foot shock: 0.7 mA, 2 s) with an interval of 1 min in a dark room (training sessions). Mice were removed from the chamber after the conditioning training. Twenty-four hours later, mice were placed back to the same chamber for 6 min in the absence of tone and shock. The amount of time with freezing behavior was recorded in a 6 s interval. Mice placed 2 h later in a test chamber that had different context and smell from the first test chamber (this second chamber was wiped with 1% acetic acid). Freezing was recorded for 3 min without the auditory conditioning stimulus. The auditory stimulus then was turned on for 3 cycles, each cycle for 30 s followed by 1-min inter-cycle interval (4.5 min in total). The freezing behavior in the 4.5 min was recorded.

### Evaluation of motor coordination

Motor coordination and exercise tolerance were evaluated two days after fear conditioning as we did before [21]. Mice were placed on an accelerating rotarod. The speed of the rotarod was increased from 4 to 40 r.p.m. in 5 min. The latency and speed of the rotarod at which a mouse fell off the rotarod were recorded. Each mouse was tested five times. The speed-latency index (latency in seconds × speed in r.p.m.) of each of the five tests was calculated, and the mean index of the five trials was used to reflect the motor coordination function and exercise tolerance of each mouse.

### Blood collection and measurement of blood chemicals

After rotarod test, mice were fasted overnight. They were deeply anesthetized by isoflurane. Blood was collected from the left ventricle of the heart and sent to the Clinical Laboratory of the University of Virginia (Charlottesville, VA) to determine lipid profiles and the levels of glucose, albumin and creatinine levels.

### Western Blot Analysis

After rotarod test, mice were sacrificed and perfused with normal saline. Total lysates of the cerebral cortex and hippocampus (50 µg protein per lane) were subjected to western blot analysis. The primary antibodies used were the mouse monoclonal anti-drebrin antibody (1∶500 dilution; catalog number: ab12350; Abcam, Cambridge, MA), rabbit polyclonal anti-BDNF antibody (1∶500 dilution; catalog number: ab6201; Abcam), the mouse monoclonal anti-NeuN antibody (1∶500 dilution; catalog number: MAB377; Millipore, Billerica, MA), the rabbit polyclonal anti-synaptophysin antibody (1∶1000 dilution; catalog number: 4329; Cell Signaling Technology Inc., Danvers, MA), the rabbit polyclonal anti-actin antibody (1∶2000 dilution; Catalog number: A2066; Sigma-Aldrich, St Louis, MO) and the rabbit polyclonal anti-glyceraldehyde 3-phosphate dehydrogenase (GAPDH) antibody (1∶2000 dilution; Catalog number: G9545; Sigma-Aldrich). The protein bands were visualized using enhanced chemiluminescence methods. To control for errors in protein sample loading and transferring during western blotting, the densities of drebrin, NeuN and BDNF protein bands were normalized to those of GAPDH and the densities of synaptophysin protein bands were normalized to those of actin because synaptophysin has a molecular weight similar to GAPDH. The results of mice in different groups were then normalized to control CD-1 wild type mice.

### Measurement of glutathione, GSSG, nitrotyrosine containing proteins and HNE

The brain samples were harvested as described for western blotting. Glutathione and GSSG levels in the cerebral cortex and hippocampus were measured with the Glutathione Assay Kit (Cayman Chemical, Ann Arbor, MI). Nitrotyrosine containing proteins and HNE in the cerebral cortex and hippocampus were assessed by the OxiSelect™ Nitrotyrosine ELISA kit (Cell Biolabs Inc., San Diego, CA) and OxiSelect™ HNE-His Adduct ELISA Kit (Cell Biolabs Inc.), respectively, according to the manufactures’ protocols. The results were normalized to the amount of proteins in the samples.

### Measurement of the thickness of hippocampal CA1 pyramidal cell layer

After rotarod test, mice were deeply anesthetized with isoflurane and perfused transcardially with saline and then 4% phosphate-buffered paraformaldehyde. Brains were removed and stored in the same fixative solution for 24 h. Four consecutive coronal sections at bregma –3.8 mm were stained by hematoxylin and eosin. Three locations, each randomly selected in the CA1 region of each side of the hippocampus in a section, were photographed and the thickness of the pyramidal cell layer was measured by an investigator who was blind to the group assignment of the animal. This measurement was aided by having a scale bar in each of the photo and performed by using the image J program (National Institutes of Health, Bethesda, MD). Total 24 measurements were performed for each animal. The average of these 24 measurements was used as the final result for the animal.

### Statistical analysis

All data in this study were parametric. They are presented as means ± S.E.M. (n ≥ 7) and analyzed by Student’s t test, Rank Sum test or by two way repeated measures analysis of variance followed by the Tukey test (for the growth curve data and the data generated during the training sessions of Barnes maze). A P<0.05 was accepted as significant. All statistical analyses were performed with the SigmaStat (Systat Software, Inc., Point Richmond, CA, USA).
